# Analyzing User Engagement Within a Patient-Reported Outcomes Texting Tool for Diabetes Management: Engagement Phenotype Study

**DOI:** 10.2196/41140

**Published:** 2022-11-14

**Authors:** Soumik Mandal, Hayley M Belli, Jocelyn Cruz, Devin Mann, Antoinette Schoenthaler

**Affiliations:** 1 Department of Population Health NYU Grossman School of Medicine New York, NY United States; 2 Technology Management and Innovation NYU Tandon School of Engineering New York, NY United States; 3 Medical Center Information Technology NYU Langone Health New York, NY United States

**Keywords:** user engagement, patient-reported outcomes, mobile health, mHealth, digital health, SMS, type 2 diabetes, health behavior, digital phenotyping

## Abstract

**Background:**

Patient-reported outcomes (PROs) capture patients’ views on their health conditions and its management, and are increasingly used in clinical trials, including those targeting type 2 diabetes (T2D). Mobile health (mHealth) tools offer novel solutions for collecting PRO data in real time. Although patients are at the center of any PRO-based intervention, few studies have examined user engagement with PRO mHealth tools.

**Objective:**

This study aimed to evaluate user engagement with a PRO mHealth tool for T2D management, identify patterns of user engagement and similarities and differences between the patients, and identify the characteristics of patients who are likely to drop out or be less engaged with a PRO mHealth tool.

**Methods:**

We extracted user engagement data from an ongoing clinical trial that tested the efficacy of a PRO mHealth tool designed to improve hemoglobin A1c levels in patients with uncontrolled T2D. To date, 61 patients have been randomized to the intervention, where they are sent 6 PRO text messages a day that are relevant to T2D self-management (healthy eating and medication adherence) over the 12-month study. To analyze user engagement, we first compared the response rate (RR) and response time between patients who completed the 12-month intervention and those who dropped out early (noncompleters). Next, we leveraged latent class trajectory modeling to classify patients from the completer group into 3 subgroups based on similarity in the longitudinal engagement data. Finally, we investigated the differences between the subgroups of completers from various cross-sections (time of the day and day of the week) and PRO types. We also explored the patient demographics and their distribution among the subgroups.

**Results:**

Overall, 19 noncompleters had a lower RR to PRO questions and took longer to respond to PRO questions than 42 completers. Among completers, the longitudinal RRs demonstrated differences in engagement patterns over time. The completers with the lowest engagement showed peak engagement during month 5, almost at the midstage of the program. The remaining subgroups showed peak engagement at the beginning of the intervention, followed by either a steady decline or sustained high engagement. Comparisons of the demographic characteristics showed significant differences between the high engaged and low engaged subgroups. The high engaged completers were predominantly older, of Hispanic descent, bilingual, and had a graduate degree. In comparison, the low engaged subgroup was composed mostly of African American patients who reported the lowest annual income, with one of every 3 patients earning less than US $20,000 annually.

**Conclusions:**

There are discernible engagement phenotypes based on individual PRO responses, and their patterns vary in the timing of peak engagement and demographics. Future studies could use these findings to predict engagement categories and tailor interventions to promote longitudinal engagement.

**Trial Registration:**

Clinicaltrials.gov NCT03652389; https://clinicaltrials.gov/ct2/show/NCT03652389

**International Registered Report Identifier (IRRID):**

RR2-10.2196/18554

## Introduction

### Background

A patient-reported outcome (PRO) is defined by the National Quality Forum and Food & Drug Administration as a “report of the status of a patient’s health condition that comes directly from the patient without amendment or interpretation of the patient’s response by a clinician or anyone else.” [1,2]. PROs include health-related quality of life [3], adherence to medical regimens, satisfaction with treatment, and elements of disease control [4]. Although innovations in medical technology have allowed the measurement of physical, physiological, or biochemical data with great accuracy, they are not able to provide the patient’s perspectives on their treatment or disease [5]. These data can only be obtained directly from patients [6]. Thus, PROs in clinical trials provide a more holistic assessment of the benefits of the treatment or intervention under investigation [7]. With the advent of patient-centered health care systems, where a patient is considered the center of the health care system [8], patients and patient advocates have called for more patient-centered outcomes reporting (ie, PROs) in combination with other clinical and physiological outcomes [5].

Traditionally, PROs have been assessed using survey instruments [4,9]. However, recent advancements in mobile health (mHealth) technologies have enabled a wide variety [10] of tools and apps that can be used to collect PRO data. With mHealth technologies, PROs can be assessed electronically from PCs, from mobile solutions such as tablet PCs or smartphones using apps or texting tools, or through data entered via web browsers [11]. The use of mHealth technologies to collect PRO data offers several advantages over traditional survey-based methods, including real-time data collection, reduced time for documentation, automated algorithms and calculations, in-home symptom monitoring, immediate transfer of data for clinical use, enhanced patient engagement in care, and more informed clinical decision-making [12-14].

Uncontrolled type 2 diabetes (T2D) is a significant public health problem in the United States, especially among vulnerable populations (eg, low-income, racial, and ethnic minorities) [15,16]. Prior studies have recognized that patients play a central role in the management of T2D (eg, being aware of its signs and symptoms and engaging in daily self-care behaviors), and several national and local organizations have launched initiatives to support the development and use of PROs in the evaluation of T2D patient care [17-21]. However, existing research that incorporates PROs in T2D care has been mostly limited to clinical drug trials examining patient tolerance to new treatment regimens [22]. The few practice-based studies conducted on T2D used long lists of PRO measures and only had patients report PROs on a single occasion, typically before clinic visits [23,24]. Such reporting increases the risk of a recall bias. To address these shortcomings, a growing number of studies are using mHealth platforms that enable real-time collection of PROs outside the clinical environment [25-31].

### Objective

Analysis of prior mHealth research revealed that most studies did not consider user engagement metrics when evaluating the design implications of the intervention on T2D patients’ health outcomes [30]. Of the few studies that reported engagement data, most were limited by small sample sizes [32] and low response rates (RRs) [33]. Although prior research has found that consideration of user preference and personalization with mHealth PRO interventions are key aspects that influence user engagement [34], there is a lack of consensus regarding best practices for modifying mHealth PRO tools to optimize digital intervention and improve patients’ engagement [35]. Thus, the ideal cadence of PRO collection to facilitate sustained engagement in an intervention is unclear and may vary according to user characteristics [36]. To address these gaps, this study reports on the analysis of longitudinal user engagement data from an ongoing randomized controlled trial (Investigating an mHealth texting tool for embedding patient-reported data in diabetes management [i-Matter]) evaluating the efficacy of a PRO mHealth texting tool for T2D management among 282 patients with uncontrolled T2D [31]. This paper discusses and compares patterns of engagement with the PRO tool among patients randomized to the i-Matter intervention and across sociodemographic characteristics to offer insights for future adaptation of the intervention based on patients’ engagement.

## Methods

### Recruitment

Patients were recruited from a network of primary care practices at NYU Langone Health across New York City’s 5 boroughs and Long Island. The details of our recruitment approach have been reported previously [31]. Briefly, to participate in i-Matter, patients must (1) have a diagnosis of T2D for ≥6 months, (2) have uncontrolled T2D defined as hemoglobin A1c >7% documented in the electronic health record (EHR) at least twice in the past year, (3) be fluent in English or Spanish, (4) be willing to send and receive text messages, and (5) be >18 years of age. Patients were excluded if they (1) refused or were unable to provide informed consent; (2) had acute renal failure, end-stage renal disease, evidence of dialysis, renal transplantation, or other end-stage renal disease–related services documented in the EHR; (3) participated in another T2D study; (4) had significant psychiatric comorbidity or reports of substance abuse (as documented in the EHR); (5) were pregnant or planning to become pregnant within 12 months; or (6) planned to discontinue care at the practice within the next 12 months.

This paper focuses on 61 patients randomized to the i-Matter intervention who have either dropped out of the trial before the 12-month study visit (ie, noncompleters) or completed the trial (ie, completers). We excluded patients who were currently participating in the trial as their data were incomplete and would not provide a comprehensive view of how their engagement with the PRO messages may change over time.

### The i-Matter Intervention

The “Investigating an mHealth texting tool for embedding patient-reported data into diabetes management” (i-Matter) trial is evaluating the efficacy of an innovative mobile PRO system that incorporates patients’ perspective of their disease into the management of T2D in primary care practices. Patients participating in the trial were randomized to the i-Matter intervention or usual care (ie, standard diabetes care by the primary care provider) in a 1:1 ratio by the study statistician. The i-Matter intervention uses text messaging to capture patients’ self-reported PROs in real time, provides data-driven feedback and motivational messages based on responses to the PROs, and creates dynamic visualizations of the PROs that are shared in personalized reports and integrated into the clinical EHR. We are currently conducting a randomized controlled trial to evaluate the efficacy of the i-Matter intervention versus usual care in reducing hemoglobin A1c levels and adherence to self-care behaviors at 12 months among 282 patients with uncontrolled T2D who receive care in resource-limited primary care practices.

### Description of PRO Messages Embedded in the i-Matter Intervention

The details of the PRO system development have been reported elsewhere [31]. Briefly, we used a mixed method, user-centered design approach to select PROs that were integrated into the i-Matter intervention. Our approach included reviewing the existing literature on PRO measures for T2D, conducting interviews with primary care providers and patients to capture their experiences with T2D, and collecting survey data. These data sources were combined to identify the PROs that would be integrated into a beta version of i-Matter and refined with user testing among patients with T2D. Table 1 lists the final set of PROs, including their timing and response options, which were integrated into the i-Matter intervention. Daily daytime messages included PROs on sleep quality and healthy eating, whereas a daily nighttime message included a PRO on physical activity (Table 1 provides further details). Patients can choose when they would like to receive medication adherence [37] PRO based on their medication regimen in the afternoon, at night, or at both times. The remaining PROs were similarly timed for all patients. In addition, patients can choose one healthy living goal from a selected list of topics identified in user testing (Table 1). Questions on individualized healthy living goals and patients’ diabetes quality of life were sent weekly, and the remaining messages were sent daily. PROs can be sent in either English or Spanish depending on the patients’ language preferences.

**Table 1:** Text (patient-reported outcome) messages in the Investigating a mobile health texting tool for embedding patient-reported data in diabetes management (i-Matter) program, their scheduled time, and accepted response.

Patient-reported outcome question or category	Timing	Valid response
Reply with the number that best describes how well you slept last night. Scale: 0 (poor)-10 (excellent) or Sleep quality	Daily at 9 AM	0-10
i-Matter (TM): Other than your regular job, did you do any physical activities like brisk walking for at least 30 minutes today? or Physical activity	Daily at 8 PM	Y, Yes (English only) S, Si, Sí (Spanish only) N, No
Have you taken all of your diabetes medications as prescribed today? or Medication adherence	Allow patients to decide if they want the message in the 1 PM or 9 PM, or both	Y, Yes (English only) S, Si, Sí (Spanish only) N, No
In general, how healthy was your overall diet yesterday? or Healthy eating	Daily at 11 AM	0-10
Custom living goal: 1=Lose weight 2=Eat more fruit or vegs 3=Eat less sweets or carbs 4=Have better portion control How successful were you in achieving your goal to [custom text healthy goal] yesterday? Healthy living goal	Weekly at 2 PM	0-10
Reply with the number that best describes how much control you felt you had over your diabetes over the past week. Scale: 0 (poor)-10 (excellent) or Quality of life	Weekly at 4 PM	0-10

### Measures

#### Engagement Metrics

User engagement data were extracted from the patients’ responses to the PROs embedded in the texting tool at the end of their participation in the 12-month study. The primary engagement metrics evaluated in this study are listed in Table 2, including the metrics of RR and response time (RT). *RR* represents the percentage of PRO questions that garnered any valid responses. The RR metric represents patient engagement with the individual PRO questions [36]. *The RR* of a message was measured as the time difference in seconds between when the PRO message was sent to a patient and the time the patient sent the corresponding response. Only valid responses to the PROs were used when measuring RT. The RT metric reflects how well the timing of PRO messages integrates into patients’ everyday lives, which in turn is expected to affect their level of engagement.

The overall RR and RT were measured by taking the average of the corresponding measures over a time frame, either weekly or monthly. We also measured RRs and RTs by grouping the messages sent at similar times of the day (daytime, nighttime) or times of the week (weekdays and weekends) and those that required similar responses (Yes or No, 1-10). Depending on the patient’s decision regarding when to receive the message, the medication adherence PRO was either used once or twice for the nighttime RR and RT measures.

**Table 2:** Engagement measures used to analyze patients’ engagement in Investigating a mobile health texting tool for embedding patient-reported data in diabetes management (i-Matter) program.

Engagement measure	Measurement or descriptions
RR^a^	Number of corresponding messages that received a valid response × 100 number of messages sent by the program with questions on PRO^b^
RT^c^	Difference between the timestamp of an incoming message sent to a patient and the timestamp of corresponding outgoing response in seconds
Weekdays RR	Number of valid corresponding responses received × 100 number of messages with questions on PRO sent between Monday and Friday
Weekdays RT	Average RT of messages responded by the patients between Monday and Friday every week
Weekends RR	Number of valid corresponding responses received × 100 number of messages with questions on PRO sent on Saturdays, Sundays
Weekends RT	Average RT of all messages responded by the patients that were sent on Saturdays and Sundays every week
Daytime messages RR	Number of valid corresponding responses received × 100 number of messages with questions on PRO sent daily at AM (before noon)
Daytime messages RT	Average RT of all messages that were sent before 11:59 AM and were responded to by the patients.
Nighttime messages RR	Number of valid corresponding responses received × 100 number of messages with questions on PRO sent daily at PM (after noon)
Nighttime messages RT	Average RT of all messages that were sent after 11:59 AM and were responded to by the patients.
Binary messages RR	Average RR of all messages for which accepted responses are Yes, Y, S, Si, Sí, or N, No.
Binary messages RT	Average RT of all messages for which accepted responses are Yes, Y, S, Si, Sí, or N, No.

^a^RR: response rate.

^b^PRO: patient-reported outcome.

^c^RT: response time.

#### Demographic Characteristics

At baseline, all patients completed a self-report instrument that was used to collect patient sociodemographic data, including sex, race or ethnicity, age, annual household income, education level, marital status, and employment status.

### Analysis

To analyze user engagement with PRO messages, we first compared the engagement metrics of RR and RT between users who completed the 12-month study (ie, completers) and those who ended their participation before program completion (ie, noncompleters). In addition, we investigated the distribution of dropout times among noncompleters. The goal of this analysis was to identify participants who were likely to drop out of the program at the early stages and tailor the program to minimize dropouts in future iterations.

We compared the longitudinal data of user engagement from the completer group using latent class trajectory modeling (LCTM). The goal was to classify heterogeneous populations into homogeneous clusters or subgroups with distinct trajectories [38] based on similarities in their engagement behaviors. For the LCTM models, we experimented with user engagement measures at various time intervals, including weekly, biweekly (measured once every 2 weeks), monthly, and bimonthly (once every 2 months). We chose the monthly engagement measures for the final analysis, as they provided a balance between smaller weekly and biweekly units, where the difference in engagement between the classes would be less distinguishable, and the larger bimonthly engagement measures that would entail smaller sample sizes for trajectories.

Finally, we investigated the difference in engagement between the subgroups identified by the LCTM model from various cross-sections of time and PRO message types. The Shapiro–Wilk normality test was used to evaluate all engagement measures for each group separately before conducting comparisons. We also explored patients’ overall sociodemographic characteristics and their distribution among the subgroups. The goal of the analyses was to further characterize the subgroups and identify patients who were likely to be part of a subgroup.

### Ethics Approval

This study was approved by the NYU Langone Health Institutional Review Board (i18-01044).

## Results

### Patient Engagement in the i-Matter Intervention

As of April 2022, a total of 61 patients *completed* their participation in the i-Matter intervention. Of the 61 participants, 42 (69%) completed the 12-month program, whereas the remaining 19 (31%) noncompleters ended their participation either by *opting out* on their own (10/61, 16%) or requesting the recruitment team to *disenroll* (9/61, 15%) from the program before the 12-month end point.

### Overall Engagement Metrics in the Noncompleters Group

**Figure 1:**
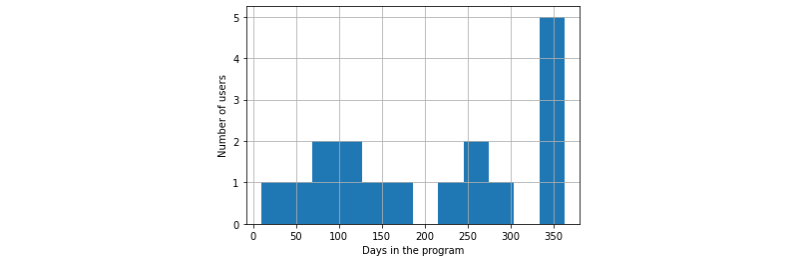
Duration in the program for patients who did not complete (noncompleters) the study.

Figure 1 shows the distribution of the time (in days) when the 19 patients ended their participation before the 12-month study visit. The average participation time in the program was 211 (SD 124.99; range 9-363) days. At least 53% (10/19) of patients dropped out of the program before the average participation time. Of the remaining patients, 4 ended participation in the last week (after 356 days from the day of enrollment) of the program. In all 4 cases, the overall RR was above 70% (mean 85.92%, SD 10.73%; median 90%, IQR 10.34%), suggesting that ending the program could have been unintended.

### Overall Engagement Metrics in the Completers Group

**Figure 2:**
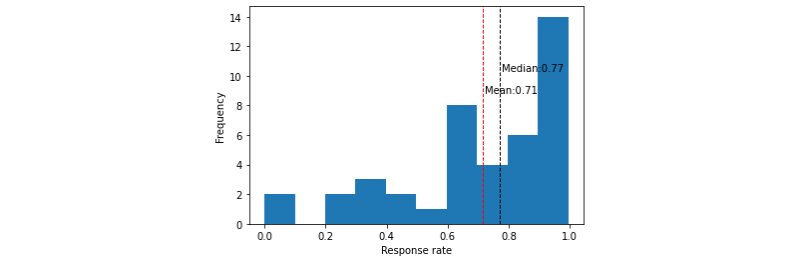
Distribution of response rate for the completers group.

The mean RR among the completers was 71.44% (SD 26.50%), and the median was 76.91% (IQR 32.72%). Figure 2 shows the distribution of RR for this group. Approximately 45% of patients had an RR below the mean value. The distribution of RRs was found to be nonnormal (*P*<.001).

### Comparison in Engagement Metrics Between the Noncompleters and Completers

**Figure 3:**
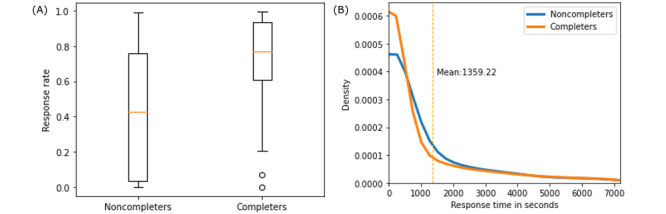
Comparison of response rate (A) and response time (B) between the completers and noncompleters group.

Figure 3 shows the comparison of PRO engagement metrics between patients who ended the program early (noncompleters) versus those who completed the entire 12 months (completers). The RR of participants (Figure 3A) who completed the program was much higher (mean 71.44%, SD 26.50%) than that of those who did not (mean 47.40%, SD 37.33%). In addition, the distributions of RTs in the 2 groups in Figure 3B suggest that completers, on average, were quicker (mean 1325, SD 3709 seconds) to respond to PRO messages than the noncompleters (mean 1359 seconds, SD 3754 seconds); however, the difference was not significant because of large variations in the RTs.

### Description of the Engagement Subgroups Among the Completers

**Figure 4:**
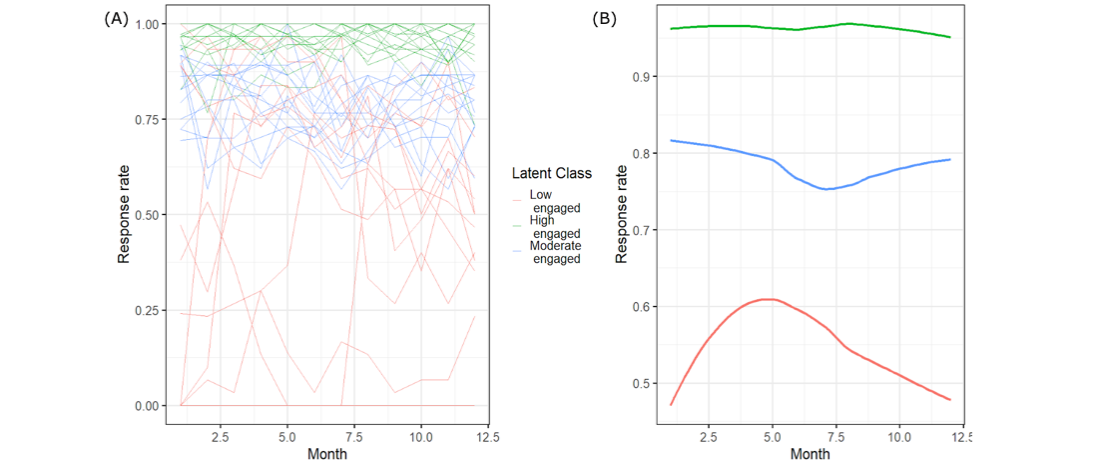
Trajectories of user engagement among completers in the Investigating an mHealth texting tool for embedding patient-reported data in diabetes management (i-Matter) intervention, (A) individual raw engagement in the left panel, (B) smoothed mean engagement in the right panel.

Initially, we constructed a scoping model that provisionally selected a plausible number of classes, *K*=2, for the LCTM. In the next step, we refined the preliminary working model by altering the number of classes (*K*=3) and exploring variations in latent class linear mixed models. We capped the number of classes at *K*=3 because of the sample size (ie, number of patients) in our study. The number of classes for the final model was determined based on the lowest Bayesian information criterion. On the basis of distribution of our outcome variable, RR in the completers group, we investigated both standard linear mixed models (using the *hlme* function) and latent process, latent class mixed models (*lcmm* function). Finally, we performed model adequacy assessments by examining the posterior probability of being assigned to each trajectory class and assigning each individual to the class with the highest probability. An average of these maximum posterior probabilities of assignments above 70% [39] in all classes was considered acceptable. We tested a total of 11 models (Multimedia Appendix 1), and in the end, a latent class linear mixed model with *K*=3 classes was chosen as the best fit. The results from the trajectories of the 3 classes are shown in Figures 4A and 4B. We defined the 3 classes as low engaged (red), moderate engaged (blue), and high engaged (green) subgroups. Spaghetti plots of individual-level data illustrate that initial RRs, combined with the timing and direction of changes in the engagement metrics, characterize the subgroups. For example, as shown in Figure 4B, the low engaged subgroup is characterized by the lowest RR at the initial weeks of the intervention, coupled with a sharp increase in RR until the midtrajectory (~5 months), followed by a steady decline in RR for the remainder of patient participation. In contrast, the RR of the moderate engaged subgroup begins above 80%, decreases at the midpoint of the intervention to 75%, and then steadily rises toward the final months to 79%. Finally, the high engaged subgroup showed a consistently high RR across the 12-month study period (Figure 4B).

### Comparison of Engagement Subgroups Among the Completers

#### Overview

Table 3 displays the RR and RT across the 3 subgroups according to the day of the week and the time of day each PRO message was sent. As the distribution of RTs was found to be significantly nonnormal for the entire population and also for the 3 subgroups individually, we used Kruskal-Wallis 1-way ANOVA to compare engagement among the 3 subgroups, followed by post hoc Dunn tests with Bonferroni adjustments for pairwise comparisons. Overall, the results suggest that patients in the high engaged subgroup had a significantly higher overall RR (96.27%) than those in the moderate engaged (78.66%) or low engaged (54.35%) subgroups (Table 4). In addition, patients in the high engaged subgroup, on average, took a significantly shorter time (mean 559 seconds, SD 451 seconds) to respond to PRO messages than the moderate engaged (1564 seconds, SD 1138 seconds) or low engaged subgroups (2814 seconds, SD 5115 seconds). The differences in engagement between the 3 subgroups were consistent across most measures, regardless of the day of the week (weekdays and weekends) or timing of the messages (daytime vs nighttime; Table 4). In addition, the RRs of messages sent at night (highlighted in gray) were found to be higher than the overall RR for all 3 subgroups.

**Table 3:** Comparisons of user engagement with text messages between the 3 subgroups (*P*<.05).

Engagement measures (RR^a^, RT^b^)^c^	Low engaged (n=12), mean (SD)	Moderate engaged (n=13), mean (SD)	High engaged (n=17), mean (SD)	Difference in distribution, *P* value
Overall RR (%)	54.35 (31.77)	78.66 (9.14)	96.27 (4.56)	<.001
Overall RT	2814 (5115)	1564 (1139)	559 (451)	<.001
Weekdays RR (%)	54.28 (31.80)	80.29 (10.50)	96.68 (4.32)	<.001
Weekdays RT	2919 (5761)	1581 (1246)	511 (445)	<.001
Weekends RR (%)	54.53 (35.41)	74.41 (16.62)	95.24 (9.35)	<.001
Weekends RT	2034 (3346)	1636 (1783)	694 (852)	<.001
Daytime messages RR (%)	48.32 (34.25)	68.22 (20.04)	93.87 (9.63)	<.001
Daytime messages RT	2814 (5115)	1564 (1139)	559 (451)	<.001
Nighttime messages RR (%)	56.27 (32.27)^d^	83.12^d^ (10.75)^d^	97.02 (4.34)^d^	<.001
Nighttime messages RT	2933 (5357)	1656 (1322)	494 (538)	<.001

^a^RR: response rate.

^b^RT: response time.

^c^All response time values are in seconds

^d^Engagement measures with greater than overall RR.

**Table 4:** Results of post hoc Dunn tests (after Kruskal-Wallis tests) on the extracted measures between the engagement subgroups.

Engagement measures	Pairwise comparison subgroups	*z-*score	*P* value	
			Unadjusted	Adjusted
**Overall RR^a^**				
	Low-high	−16.92	<.001	<.001
	Low-moderate	−3.84	<.001	<.001
	Moderate-high	13.14	<.001	<.001
**Overall RT^b^**				
	Low-high	8.24	<.001	<.001
	Low-moderate^c,d^	−0.82	.41	.97
	Moderate-high	−9.33	<.001	<.001
**Weekdays RR**				
	Low-high	−16.68	<.001	<.001
	Low-moderate	−4.74	<.001	<.001
	Moderate-high	11.92	<.001	<.001
**Weekdays RT**				
	Low-high	7.87	<.001	<.001
	Low-moderate^c,d^	−1.02	.31	.92
	Moderate-high	−9.16	<.001	<.001
**Weekends RR**				
	Low-high	−13.71	<.001	<.001
	Low-moderate	−2.73	<.01	<.05
	Moderate-high	11.06	<.001	<.001
**Weekends RT**				
	Low-high	3.13	<.01	<.01
	Low-moderate	−2.48	<.05	<.05
	Moderate-high	−5.90	<.001	<.001
**Daytime messages RR**				
	Low-high	−14.42	<.001	<.001
	Low-moderate	−3.17	<.01	<.01
	Moderate-high	11.32	<.001	<.001
**Daytime messages RT**				
	Low-high	8.24	<.001	<.001
	Low-moderate^c,d^	−0.82	.41	.90
	Moderate-high	−9.33	<.001	<.001
**Nighttime messages RR**				
	Low-high	−16.62	<.001	<.001
	Low-moderate	−5.24	<.001	<.001
	Moderate-high	11.32	<.001	<.001
**Nighttime messages RT**				
	Low-high	8.15	<.001	<.001
	Low-moderate^c,d^	−1.28	.20	.61
	Moderate-high	−9.72	<.001	<.001

^a^RR: response rate.

^b^RT: response time.

^c^No significant difference before adjustment.

^d^No significant difference after adjustment.

We also compared user engagement between subgroups for the 6 PRO messages individually. In addition, the PROs were grouped by response type (yes or no as binary, and the remaining messages as a Likert-type scale) and compared separately. As shown in Table 5, a significant difference in the RR was observed for all 6 messages. For example, the mean RRs of sleep quality PRO were 44.84%, 60.44%, and 93.28% in the low engaged, moderate engaged, and high engaged subgroups, respectively. Similarly, RT was also significantly different among the 3 subgroups for all messages, except for physical activity. Overall, the binary PROs (Yes or No) had a higher RR than the Likert-scale PROs for all 3 subgroups. In fact, patients’ RR to binary PRO messages, medication adherence, and diet (healthy eating) were above average for all 3 subgroups. In contrast, patients were consistently less responsive to the Likert-scale PRO on fulfilling a healthy living goal (weekly at 2 PM), with only one of 3 messages receiving any valid response from the low engaged subgroup. Further details of pairwise comparisons of the subgroups are provided in Table 6.

**Table 5:** User engagement and patient-reported outcome message types between the 3 subgroups (*P*<.05).

Engagement measures (RR^a^, RT^b^)	Low engaged (n=12), mean (SD)	Moderate engaged (n=13), mean (SD)	High engaged (n=17), mean (SD)	Difference in distribution, *P* value
Binary messages RR (%)	57.13 (34.50)	82.93 (11.41)	97.19 (5.14)	<.001
Binary messages RT	3191 (7354)	1630 (1576)	578 (715)	<.001
Likert-scale messages RR (%)	51.12 (31.22)	74.32 (13.11)	95.31 (6.48)	<.001
Likert-scale messages RT	2056 (2797)	1504 (1424)	563 (461)	<.001
Sleep quality RR (%)	44.84 (33.39)	60.44 (22.94)	93.28 (10.17)	<.001
Sleep quality RT	1353 (1500)	1568 (1331)	946 (765)	<.001
Physical activity RR (%)	45.54 (35.32)	70.82 (19.36)	94.95 (9.34)	<.001
Physical activity RT	1183 (1304)	1125 (855)	1005 (1084)	.07
Medication adherence RR (%)	65.03 (36.67)^c^	91.68 (10.93)^c^	98.84 (4.00)^c^	<.001
Medication adherence RT	3097 (7476)	1800 (2256)	250 (865)	<.001
Healthy eating RR (%)	60.23 (34.33)^c^	88.29 (13.42)^c^	98.31 (5.63)^c^	<.001
Healthy eating RT	1867 (3358)	1373 (2073)	195 (503)	<.001
Healthy living goal RR (%)	33.33 (47.30)	64.10 (48.12)	91.18 (28.43)	<.001
Healthy living goal RT	444 (1243)	710 (1260)	772 (1356)	<.001
Quality of life RR (%)	50 (50.17)	85.26 (35.57)	92.65 (26.16)	<.001
Quality of life RT	1187 (2977)	1487 (3518)	250 (1125)	<.001

^a^RR: response rate.

^b^RT: response time.

^c^Patient-reported outcome messages with greater than average response rate.

**Table 6:** Results of post hoc Dunn tests (after Kruskal-Wallis tests) on the patient-reported outcome message types between the engagement subgroups.

Engagement measures	Pairwise comparison subgroups	*z*-score	*P* value	
			Unadjusted	Adjusted
**Binary messages RR^a^**				
	Low-high	−15.41	<.001	<.001
	Low-moderate	−4.29	<.001	<.001
	Moderate-high	11.12	<.001	<.001
**Binary messages RT^b^**				
	Low-high	6.39	<.001	<.001
	Low-moderate^c,d^	−1.72	.08	.26
	Moderate-high	−8.40	<.001	<.001
**Likert-scale messages RR**				
	Low-high	−15.91	<.001	<.001
	Low-moderate	−4.16	<.001	<.001
	Moderate-high	11.76	<.001	<.001
**Likert-scale messages RT**				
	Low-high	6.13	<.001	<.001
	Low-moderate^c,d^	−1.08	.28	.85
	Moderate-high	−7.44	<.001	<.001
**Sleep quality RR**				
	Low-high	−14.54	<.001	<.001
	Low-moderate	−2.63	<.01	<.05
	Moderate-high	12.02	<.001	<.001
**Sleep quality RT**				
	Low-high^c,d^	1.09	.28	.83
	Low-moderate	−3.09	<.01	<.01
	Moderate-high	−4.47	<.001	<.001
**Physical activity RR**				
	Low-high	−14.80	<.001	<.001
	Low-moderate	−4.22	<.001	<.001
	Moderate-high	10.57	<.001	<.001
**Physical activity RT**				
	Low-high^c,d^	0.12	.90	.99
	Low-moderate^c,d^	−1.83	.06	.19
	Moderate-high^d^	−2.11	.03	.10
**Medication adherence RR**				
	Low-high	−13.01	<.001	<.001
	Low-moderate	−6.16	<.001	<.001
	Moderate-high	6.62	<.001	<.001
**Medication adherence RT**				
	Low-high	7.56	<.001	<.001
	Low-moderate^c,d^	−2.65	.40	. 32
	Moderate-high	−10.61	<.001	<.001
**Healthy eating RR**				
	Low-high	−14.30	<.001	<.001
	Low-moderate	−6.94	<.001	<.001
	Moderate-high	7.09	<.001	<.001
**Healthy eating RT**				
	Low-high	7.35	<.001	<.001
	Low-moderate^c,d^	−1.93	.054	.16
	Moderate-high	−9.62	<.001	<.001
**Healthy living goal RR**				
	Low-high	−11.23	<.001	<.001
	Low-moderate	−5.63	<.001	<.001
	Moderate-high	5.38	<.001	<.001
**Healthy living goal RT**				
	Low-high	−8.26	<.001	<.001
	Low-moderate	−5.17	<.001	<.001
	Moderate-high	2.84	<.01	<.05
**Quality of life RR**				
	Low-high	−9.48	<.001	<.001
	Low-moderate	−7.38	<.001	<.001
	Moderate-high^c,d^	1.68	.09	.27
**Quality of life RT**				
	Low-high^c,d^	−1.82	.07	.20
	Low-moderate	−4.27	<.001	<.001
	Moderate-high	−2.78	<.01	<.05

^a^RR: response rate.

^b^RT: response time.

^c^No significant difference (*P*<.05) before adjustment.

^d^No significant difference after adjustment.

#### Comparison of Patient Sociodemographic Characteristics Among the Engagement Subgroups

We examined patients’ sociodemographic characteristics for those who completed the program (n=42) and compared their distribution among the 3 engagement subgroups. In 6.85% (23/336) of cases, at least one of the sociodemographic questionnaires was missing responses. As shown in Table 7, the overall patient sample was mostly female (12/42, 71%), non-Hispanic or Latino origin (22/28, 79%), fluent in English (38/41, 93%), completed a bachelor’s degree or above (21/41, 52%), and married or living with a partner (18/41, 44%). The distribution of demographic data further shows that relative to the overall population distribution, a higher proportion of male Hispanic or Latino origin patients were more high engaged than females and non-Hispanic or Latino origin counterparts. In addition, the high engaged subgroup was composed of mostly older patients with the lowest variations in age range (between 53 and 68 years), and a higher percentage of Hispanic or Latino patients (3/10, 30%) were bilingual (2/16, 13%), and completed at least a graduate-level education (5/16, 31%).

In comparison, the low engaged subgroup was composed primarily of patients who were identified as African Americans (7/12, 58%) and reported the lowest annual income, with one out of every 3 patients earning US $20,000 or less annually. Finally, marital status was relatively consistent between low-engaged and high-engaged groups.

**Table 7:** Summary of participants’ demographics.

Demographics			Overall (n=42)		Low engaged		Moderate engaged		High engaged
**Gender, n (%)**									
	Male	12 (29)		3 (25)		4 (31)		5 (29)	
	Female	30 (71)		9 (75)		9 (69)		12 (71)	
	Other or missing	0 (0)		0 (0)		0 (0)		0 (0)	
**Age (years)**									
	Range	32-73		45-73		32-68		53-68	
	Mean (SD)	59.30 (8.37)		59.92 (8.37)		56.33 (9.92)		61.06 (4.23)	
**Race, n (%)**									
	African American	20 (48)		7 (58)		6 (46)		7 (41)	
	White	13 (31)		2 (17)		5 (38)		6 (35)	
	Other races	3 (7)		2 (17)		1 (8)		0 (0)	
	Refused	1 (2)		0 (0)		0 (0)		1 (6)	
	Unknown	5 (12)		1 (8)		1 (8)		3 (18)	
**Ethnicity, n (%)**									
	Hispanic or Latino	6 (21)		1 (12)		2 (20)		3 (30)	
	Not Hispanic or Latino	22 (79)		7 (88)		8 (80)		7 (70)	
	Missing	14		4		3		7	
**Language, n (%)**									
	English	38 (93)		11 (92)		13 (100)		14 (88)	
	Spanish	1 (2)		1 (8)		0 (0)		0 (0)	
	Both	2 (5)		0 (0)		0 (0)		2 (12)	
	Missing	1		0		0		1	
**Education, n (%)**									
	Graduate	11 (27)		3 (25)		3 (23)		5 (31)	
	Bachelor	10 (25)		4 (34)		3 (23)		3 (19)	
	Associate	2 (5)		0 (0)		1 (8)		1 (6)	
	Some college or no degree	12 (29)		3 (25)		5 (38)		4 (25)	
	Technical school	12 (29)		1 (8)		0 (0)		0 (0)	
	High school or General Educational Development	4 (10)		1 (8)		1 (8)		2 (13)	
	Grades 1-8	1 (2)		0 (0)		0 (0)		1 (6)	
	Missing	1		0		0		1	
**Income per year, n (%)**									
	<US $10,000	1 (3)		1 (10)		0 (0)		0 (0)	
	<US $20,000	5 (14)		3 (30)		1 (9)		1 (7)	
	<US $40,000	10 (28)		4 (40)		2 (18)		4 (27)	
	<US $60,000	9 (24)		2 (20)		2 (18)		5 (33)	
	≤US $100,000	10 (28)		0 (0)		5 (46)		5 (33)	
	>US $100,000	1 (3)		0 (0)		1 (9)		0 (0)	
	Missing	6		2		2		2	
**Marital status, n (%)**									
	Married or partner	18 (44)		5 (42)		7 (54)		7 (44)	
	Never married	14 (34)		4 (33)		4 (30)		5 (31)	
	Divorced	6 (15)		2 (17)		1 (8)		3 (19)	
	Windowed	3 (7)		1 (8)		1 (8)		1 (6)	
	Missing	1		0		0		1	

## Discussion

### Principal Findings

Although achieving glycemic control is of clinical importance for patients with T2D, it is the daily experience of living with T2D that drives patients’ perseverance to adhere to treatment regimens and become engaged in their care [40]. This study reports user engagement with PROs in the i-Matter trial, designed to incorporate the collection of real-time PRO data that are meaningful to both patients and providers in the clinical management of T2D. Our retrospective analysis of user engagement found discernible engagement phenotypes based on individual PRO responses: patients who dropped out from the program early (noncompleters) took more time to respond to PRO questions and were less likely to respond than the completers. Among the completers, the analysis of the longitudinal RRs identified 3 subgroups with significant differences in engagement. The completers from the lowest engagement subgroup had a significantly lower RR and longer RT than those from the other 2 completer subgroups. These results suggest that patients who have lower RR in combination with longer RTs are at risk of dropping out of the program or continuing with lower than average engagement with PRO questions. Future analyses will evaluate whether this pattern is associated with poorer adherence to self-management behaviors.

Our analysis further revealed that the engagement phenotypes among completers differed in the timing of peak engagement. The low engaged completers showed an almost normal distribution of average engagement over time, with peak engagement in the middle of the program, followed by a steady decline. The decline in engagement following the peak could be due to fatigue onset among patients from responding to multiple daily text messages across the 12-month study [41]. Further evaluation and analysis are required to determine how to maintain peak engagement in this subgroup in the latter half of the program. The moderate engaged subgroup showed an opposite trend in peak engagement compared with the low engaged subgroup: a decline in engagement in the first 7 months of the program, followed by a steady increase.

The high engaged subgroup consistently showed >90% RR and low RT throughout their participation. They also represent the largest sample among the 3 subgroups. The reasons for high engagement and whether this response pattern leads to better health outcomes (behavioral and clinical) among patients with T2D will be explored in future analyses. The variations in peak engagement timing suggest that longitudinal data on patients’ motivations and self-care behavioral activities need to be analyzed for periodic changes and to evaluate their impact on user engagement.

Traditionally, sociodemographic characteristics, including older age, lower income, unemployment status, lower education status, minority racial and ethnic group membership, language barriers, and geographic barriers, have been associated with an elevated risk for poorer health outcomes [42]. Our analysis of the sociodemographic measures also showed that distributions of race, age, language, and income varied between the high- and low engaged subgroups of our intervention. The high engaged completers were predominantly older, of Hispanic descent, bilingual, and highly educated (graduate school or above).

These findings reflect the growing trends in mHealth research, which has shown that behavioral interventions, particularly those that leverage text messaging, have high rates of user engagement among Hispanic individuals with limited English proficiency [43,44]. For example, Cartujano-Barrera et al [44] reported high levels of interactivity (73%) and low disenrollment (20%) with a 12-week smoking cessation intervention delivered via text messaging in a sample of bilingual Hispanic individuals. Similarly, a text messaging intervention designed to improve diabetes management showed high levels of engagement (86% RR to at least one text message) and low dropout rates (6%) among low-income Hispanic patients who were followed in safety-net primary care practices [45]. This is in line with data published by the Pew Research Center [46], which showed higher cell phone ownership and use among Hispanic than among non-Hispanic White patients (100% vs 97%). Data from qualitative evaluations of text messaging studies in Spanish-speaking Hispanic individuals suggest that receiving and responding to text messages serves as a source of emotional support [45] for engaging in self-care behaviors to improve diabetes management. Prior research on disparities in self-care behaviors among patients with T2D reported consistent evidence of no disparities in exercise and some evidence of reverse disparities. Compared with non-Hispanic White patients, Hispanic patients with T2D had healthier diets [47], which likely manifested in higher engagement, especially with Healthy Eating PRO among Hispanic patients. Although our finding that older adults were more likely to be high engagers of the intervention may seem inconsistent with previous research [48-50], recent data show that more than 85% of adults aged ≥50 years communicate primarily via text messages [51]. Research on barriers to diabetes medication adherence has also found evidence that younger age is associated with motivational and behavioral barriers [37], which could have manifested in lower engagement among younger patients in our intervention.

Our analysis further suggests that African American patients with the lowest annual income are most likely to have low engagement with PRO messages. It is plausible that the initial development of our intervention may not have captured the unique needs and concerns of this patient population. Consequently, the content and delivery of messages may not have been suitable, resulting in low engagement rates. Previous research has also found barriers to low-income African American patients’ engagement in text messaging interventions, including low ownership of personal cell phones, difficulty in responding to text messages, and reporting that the program was not helpful or relevant for improving self-management behaviors [37]. These findings suggest that future text message interventions must consider both the technical elements of the intervention as well as the contextual factors (eg, financial and cultural) that could affect user engagement.

### Limitations

Although this study has many strengths, we note the following limitations that can be considered for future research. First, although our intervention enrolled patients with T2D, it is more common for patients to have 2 or more chronic diseases (ie, multimorbidity) than one disease in isolation (89.3% vs 8.5%, respectively) [52]. Recent research has demonstrated the negative impact of multimorbidity on PROs such as quality life, psychosocial health, self-efficacy, physical function, and self-management behaviors [53]. Thus, future research should examine whether adapting i-Matter for a multimorbid population would improve the engagement of patients and provider management of co-occurring chronic diseases rather than using a single disease focus that can cause inefficiencies and fragmentation in care. Second, we did not examine psychosocial factors that could impact patient participation in the intervention. Their motivation, knowledge, and self-efficacy behaviors before joining the program may affect their engagement with PROs [54]. Future analysis of i-Matter data will examine how user engagement metrics differ based on self-reported diabetes self-care behaviors, medication adherence, self-efficacy, and motivation for diabetes management. We also note a possible limitation of our data analysis because of the small sample size. A larger sample size would provide greater statistical power for detecting statistically significant differences in user engagement between demographics if there are true differences. Future analyses of i-Matter data will include the remaining patients who completed the trial.

### Conclusions

In summary, we found that patients who dropped out from the intervention early (ie, noncompleters) had a lower RR to PRO questions and took longer to respond to PRO questions than completers. Among completers, analysis of longitudinal RRs identified 3 subgroups of engagement over time. Our results suggest that patient sociodemographics along with RT measures offer good predictability for patients who need further support to stay engaged in the intervention. Future trials should identify these patients early in the intervention and customize PRO messages and their timing to elicit higher engagement.

The i-Matter intervention was designed and developed through active involvement of patients and addresses difficulties with protocol compliance, lack of clinical integration in the EHR, and provider skepticism about the utility of PROs in practice, which were hallmarks of previous trials, thus increasing the likelihood of developing a sustainable approach [55]. Despite these efforts, the results showed that 31.1% of patients dropped out of the intervention before the final 12-month study visit, suggesting room for improvement in the PRO texting tool for future trials. For example, the messages sent during the daytime yielded the lowest engagement from this subgroup, which suggests that further customization of PRO message timing is needed to ensure that the cadence of messages fits within the daily lives of users to increase engagement. This analysis provides insights into how to make PROs more patient-centric in future iterations of the i-Matter intervention. The current research conducting qualitative interviews with patients who complete the program will be used to identify potential motivators that could be integrated into future versions of i-Matter.

## Appendix

Multimedia Appendix 1 List of models tested to derive the user engagement classes.

## Abbreviations

EHR: electronic health record
i-Matter: Investigating an mHealth texting tool for embedding patient-reported data in diabetes management
mHealth: mobile health
LCTM: latent class trajectory modeling
PRO: patient-reported outcome
RR: response rate
RT: response time
T2D: type 2 diabetes
