# Supplementary material for: Analyzing User Engagement Within a Patient-Reported Outcomes Texting Tool for Diabetes Management: Engagement Phenotype Study
Source: JMIR Diabetes. 2022 Nov 14;7(4):e41140. doi: 10.2196/41140 (PMC9706388; doi:10.2196/41140)
Supplement: Multimedia Appendix 1 [file diabetes_v7i4e41140_app1.docx]

**Table A.1**: List of models tested to derive the user engagement classes. Final model used for the rest of the study is highlighted in gray. [RR: monthly response rate, *hlme*: latent class linear mixed model, *lcmm*: latent process mixed model]

| **Model parameters** | | | | **Evaluation parameters** | | |
| --- | --- | --- | --- | --- | --- | --- |
| IVs | Model Type | Link function | No. of class | Log likelihood | AIC | BIC |
| RR | *hlme* | BetaCDF | 2 | 309.57 | -599.14 | -582.51 |
| RR | *hlme* | BetaCDF | 3 | 317.67 | -607.34 | -584.05 |
| RR, RT | *hlme* | BetaCDF | 2 | 311.55 | -601.10 | -582.81 |
| RR, RT | *hlme* | BetaCDF | 3 | 318.37 | -606.74 | -581.79 |
| RR | *lcmm* | BetaCDF | 2 | -1.00e+09 | 2.00e+09 | 2.00e+09 |
| RR | *lcmm* | BetaCDF | 3 | -1.00e+09 | 2.00e+09 | 2.00e+09 |
| RR | *lcmm* | 2-quant splines | 2 | 5.84e+02 | -1.14e+03 | -1.12e+03 |
| RR | *lcmm* | 2-quant splines | 3 | 5.87e+02 | -1.14e+03 | -1.11e+03 |
| RR, RT | *lcmm* | BetaCDF | 2 | -1.00e+09 | 2.00e+09 | 2.00e+09 |
| RR, RT | *lcmm* | BetaCDF | 3 | -1.00e+09 | 2.00e+09 | 2.00e+09 |
| RR, RT | *lcmm* | 2-quant splines | 3 | 5.88e+02 | -1.14e+03 | -1.11e+03 |
